# Antifouling potential of Nature-inspired sulfated compounds

**DOI:** 10.1038/srep42424

**Published:** 2017-02-13

**Authors:** Joana R. Almeida, Marta Correia-da-Silva, Emília Sousa, Jorge Antunes, Madalena Pinto, Vitor Vasconcelos, Isabel Cunha

**Affiliations:** 1 CIIMAR/CIMAR - Interdisciplinary Centre of Marine and Environmental Research, University of Porto, Terminal de Cruzeiros do Porto de Leixões Avenida General Norton de Matos P 4450-208 Matosinhos, Portugal; 2 Laboratory of Organic and Pharmaceutical Chemistry, Department of Chemical Sciences, Faculty of Pharmacy, University of Porto, Rua Jorge Viterbo Ferreira, 228, 4050-313 Porto, Portugal; 3 Department of Biology, Faculty of Sciences, University of Porto, Rua do Campo Alegre, P 4069-007 Porto, Portugal

## Abstract

Natural products with a sulfated scaffold have emerged as antifouling agents with low or nontoxic effects to the environment. In this study 13 sulfated polyphenols were synthesized and tested for antifouling potential using the anti-settlement activity of mussel (*Mytilus galloprovincialis*) plantigrade post-larvae and bacterial growth inhibition towards four biofilm-forming bacterial strains. Results show that some of these Nature-inspired compounds were bioactive, particularly rutin persulfate (2), 3,6-bis(*β*-D-glucopyranosyl) xanthone persulfate (6), and gallic acid persulfate (12) against the settlement of plantigrades. The chemical precursors of sulfated compounds **2** and **12** were also tested for anti-settlement activity and it was possible to conclude that bioactivity is associated with sulfation. While compound **12** showed the most promising anti-settlement activity (EC_50_ = 8.95 μg.mL^−1^), compound **2** also caused the higher level of growth inhibition in bacteria *Vibrio harveyi* (EC_20_ = 12.5 μg.mL^−1^). All the three bioactive compounds **2**, **6**, and **12** were also found to be nontoxic to the non target species *Artemia salina* (<10% mortality at 250 μM) and *Vibrio fischeri* (LC_50_ > 1000 μg.mL^−1^). This study put forward the relevance of synthesizing non-natural sulfated small molecules to generate new nontoxic antifouling agents.

The process of biofouling involves the attachment of a range of micro- and macroorganisms in natural and artificial underwater surfaces, constituting a diverse settled community that causes serious problems and large investments to maritime industry worldwide^1^,^2^,^3^. Short-term prevention of biofouling currently implies the use of biocide-based antifouling paints. After the ban of tributyltin (TBT) in several countries^4^, some booster biocides have been introduced based on Cu^2+^ paints as less toxic antifouling (AF) agents. However, significant environmental harm was also attributed to these AF active principals^5^. Recent international regulation (EU Regulation n° 528/2012) has been issued for biocides currently used in antifouling coatings, and many have been banned by individual initiative of many countries, through tight legislation.

Considering this, efforts have been applied on the development of alternative nontoxic and environmentally friendly AF agents. These agents should be capable to inhibit the settlement of selected biofouling species by acting in more specific signaling targets, somehow related with settlement processes, instead of inducing general toxicity^6^. Therefore, in the pursuit of an environmental friendly strategy for marine biofouling control, both effectiveness and toxicity of new AF agents need to be well established.

Sulfation of biomolecules is a metabolic strategy used by Nature to prevent toxicity in different physiological and pathological processes^7^. Some marine sulfated secondary metabolites, namely sulfated polyphenols, such as flavonoids, coumarins, cinnamic acids, and sulfated sterols, have been emerged as antifoulants with low or nontoxic effects to the environment^8^,^9^,^10^,^11^,^12^,^13^,^14^. Particularly, sulfated cinnamic acid (thereafter referred as zosteric acid), a natural metabolite from the sea grass *Zostera marina*, has been mentioned as a fully biodegradable and nontoxic natural AF agent^14^,^15^,^16^,^17^. Considering that commercial supply issues limit an effective implementation of natural products in AF coatings^18^,^19^, the aim of this study was to search for potential synthetic sulfated derivatives (Fig. 1, **1**–**13**) as new relevant nontoxic AF agents. Different chemical classes were selected for sulfation (Fig. 1**1**–**13**) to represent the chemical diversity found in known marine antifoulants, namely xanthones^20^, flavonoids, including flavonols and one flavone^11^, coumarins^12^, and cinnamic acid derivatives^14^,^15^,^16^,^17^. This chemical diversity will allow structure-activity relationship (SAR) studies. Polysaccharides are a promising material for antifouling surfaces because their chemical composition makes them highly hydrophilic and able to form water-storing hydrogels. A number of recent studies showed that coatings with amphiphilic properties have a high potential for inert surface coatings, a property that can be established in the hydrophilic polysaccharides network via chemical modifications with hydrophobic molecules^21^. Therefore, flavonoids, xanthones, coumarins and other polyphenols with *C*-glysosyl and *O*-glycosyl linked to a diversity of saccharidic units, namely glucose, galactose, and rutinose were selected. The linkage between these two molecular moieties was also planned to contain a triazole group in order to mimic potent triazole-based biocides^22^. Furthermore, in order to develop an attractive protection against fouling from a bioenergetic point of view, a low cost synthesis of the potential biologically active compounds was guaranteed by the selection of raw materials such as diosmin, hesperidin, rutin, ethoxyrutin, resveratrol glucoside, gallic acid, and chlorogenic acid, which are readily available.

To assess their AF effectiveness and toxicity, ecotoxicological bioassays towards the macrofouling organism *Mytilus galloprovincialis* using adhesive plantigrades and also antibacterial assays using several biofilm-forming marine bacteria (*Cobetia marina, Vibrio harveyi, Pseudoalteromonas atlantica* and *Halomonas aquamarina*) were conducted. *Mytilus* spp. are among the most common biofouling species with worldwide representatives. The larval phases responsible for the initial settlement of *Mytilus galloprovincialis* have produced consistent results in anti-settlement bioassays^23^,^24^,^25^,^26^,^27^. Marine bacterial biofilms are known to modulate in some degree the succession of colonization of macrofouling species^28^. Evidences show that the inhibition/induction of settlement is dependent on the nature of the bacterial biofilms regarding the production/absence of proteolytic enzymes^29^. Thus, biofilm inhibition assessment of different biofilm-forming marine bacteria can give valuable insights on the effectiveness and eventually mode of action of promising bioactive compounds.

Finally, compounds showing promising AF bioactivity were further tested in complementary bioassays to assess the viability of selected compounds as AF products. These complementary bioassays include: toxicity to sensitive non target species *Artemia salina* and *Vibrio fischeri*; the assessment of the anti-settlement activity of their chemical precursors (non-sulfated) to evaluate the influence of the sulfate groups in the AF activity; the bioaccumulation potential and the evaluation of possible mechanims of action related with adhesion and neurotransmission pathways.

## Results

### Syntheses and structure elucidation of sulfated compounds

Sulfated compounds **1–13** were obtained by reaction of the corresponding hydroxylated derivatives with triethylamine-sulfur trioxide adducts in moderate yields. For the new described derivatives, 3,7-di(*β*-D-glucopyranosyl)flavone persulfate (**4**), 3,6-bis(1-(1-(*β*-D-glucopyranosyl)-1*H*-1,2,3-triazole-4-yl)methoxy)xanthone persulfate (**7**), and 4-methylumbelliferyl 7-*β*-D-galactopyranoside persulfate (**11**), microwave assisted synthesis was successful in achieving persulfation. The complete structure elucidation of the new sulfated derivatives (**4**, **7**, and **11**) was possible to obtain by IR, NMR, and HRMS. The IR spectra of sulfated derivatives showed the presence of two strong bands corresponding to the S = O (ca 1250–1260 cm^−1^) and C-O-S (ca 1030–1070 cm^−1^) and a band around 800 cm^−1^ corresponding to the S-O. The HRMS allowed the elucidation of the number of sulfate groups. The assignments of the protonated carbons were achieved from HSQC experiments and the chemical shifts of the carbon atoms not directly bonded to proton atoms were deduced from HMBC correlations. The purity of the investigated compounds **1–13** was at least 95%.

### Antifouling bioactivity

From the series of sulfated derivatives **1–13**, four compounds (**1**, **2**, **6**, and **12**) showed a significant inhibitory effect (*p* < 0.05) against the settlement of *M. galloprovincialis* plantigrades, and nine compounds were not significantly different from the negative control (Fig. 2). However, since compound **1** showed significant anti-settlement activity only for the highest concentration tested (250 μM), only three promising AF compounds (**2**, **6**, and **12**) were considered from this primary screening based on the significant anti-settlement responses at 50 μM (Fig. 2).

The concentration-response analysis using successive concentrations between 1.56–500 μM (Fig. 3) revealed that the most effective anti-settlement activity was observed for compound **12** (EC_50_ = 17.65 μM; 8.4 μg.mL^−1^), followed by compounds **2** (EC_50_ = 22.59 μM; 36.84 μg.mL^−1^) and **6** (EC_50_ = 23.19 μM; 31.74 μg.mL^−1^) (Table 1).

Compound **12** showed significant anti-settlement activity in the range 12.5–500 μM when compared to negative control, while compounds **2** and **6** only showed significant bioactivity for higher concentrations (25–500 μM) (Fig. 3).

Regarding toxicity, none of the three selected compounds caused mortality to the target species *M. galloprovincialis* plantigrades at any of the tested concentrations, while the commercial eco-friendly AF agent ECONEA^®^ showed some toxicity at the higher concentrations tested (LC_50_ = 107.78 μM) (Table 1). Therefore, LC_50_ values for sulfated compounds were considered as higher than 500 μM, and this value was used to estimate the therapeutic ratios LC_50_/EC_50_. Therapeutic ratios higher than 22.13, 21.56, and 26.61 μM were found for compounds **2**, **6**, and **12**, respectively (Table 1).

From the obtained chromatograms (data not shown), a concentration of at least 95% of the initial one was detected for each compound, **2**, **6**, and **12**, for the time period and temperature of the AF assay, warranty that the sulfated derivatives were stable in AF assay conditions.

Chemical precursors of compounds **2** and **12**, rutin (**14**) and gallic acid (**15**) respectively, did not show significant anti-settlement activity when compared to the negative control (S1).

### Acetylcholinesterase and tyrosinase activities

Acetylcholinesterase and tyrosinase activities were not inhibited by any of the three promising sulfated compounds **2**, **6**, and **12** (S2).

### Antibacterial activity

Two compounds, **2** and **4**, from the series of sulfated derivatives **1–13** showed a significant inhibitory activity against bacterial growth, namely against *V. harveyi* and *H. aquamarina* strains, respectively. The other eleven compounds either induced or had no effect on the bacterial growth when compared to the negative control (Fig. 4).

Regarding the concentration-response analysis of the two bioactive antibacterial compounds, compound **2** showed significant antibacterial activity against *V. harveyi* for the concentrations of 3.12 (27.9% growth inhibition) to 100 μM (15% growth inhibition) when compared to negative control, while compound **4** showed significant bioactivity against *H. aquamarina* from the concentrations of 12.5 (15.3% growth inhibition) to 100 μM (26.5% growth inhibition) (Fig. 5).

The percentages of growth inhibition reached, even at the higher concentrations tested, are below 30%. Thus, for both compounds the antibacterial efficacy only enabled the determination of the response concentrations that inhibited 20% of bacterial growth (EC_20_), being 7.69 and 42.3 μM (12.5 and 51.22 μg.mL^−1^) for compounds **2** and **4**, respectively (Table 2).

### *Artemia salina* ecotoxicity assay

The three compounds selected from the initial screening as potential AF compounds (**2**, **6**, and **12**) showed less than 10% mortality to *A. salina* nauplii at concentrations of 25, 50, and 250 μM (S1). The observed lethality was not significantly different (*p* < 0.01) from the negative control (filtered seawater) and thus these compounds showed no toxicity to this non target species (S3).

### Luminescent *Vibrio fischeri* ecotoxicity assay (ISO11348)

Results from the Luminescent *Vibrio fischeri* assay revealed that the three compounds selected from the initial AF screening (**2**, **6**, and **12**) do not exert significant general ecotoxicity, as no inhibition of light radiation emitted was found, either after a contact time of 15 min or 30 min (LC_50_ > 1000 μg/mL) (S4).

### *In silico* evaluation of bioaccumulation potential

In contrast to commercial AF agents, namely the toxic agent tributyltin oxide (TBTO), and two eco-friendly agents Sea-nine and ECONEA^®^, log *K*_ow_ lower than 3 were obtained for compounds **2**, **6**, and **12** (S5), indicating the low bioaccumulation potential of the three hit compounds of the present study.

## Discussion

Nature uses sulfation mainly to avoid potential toxicity, so synthesis of non-natural sulfated small molecules could be a way of giving Nature a helping hand in generating new nontoxic bioactive agents. Thus, in this work, a series of Nature inspired sulfated compounds **1–13** was synthesized and tested for the AF potential. This series of compounds was obtained by a simple and efficient synthesis, with yields exceeding 80%, with high scale-up potential, isolated with purity higher than 95% by a purification process under environmentally favorable conditions (Portuguese patent n° 104739)^30^.

From the AF screening, persulfated flavonoid **2**, xanthone **6**, and phenolic acid **12**, showed therapeutic ratios (LC_50/_EC_50_) higher than 15, which is in accordance with the standard requirement for efficacy level of natural AF agents as established by the US Navy program^31^. Since chemical precursors tested, rutin (**14**) and gallic acid (**15**), were not active towards anti-settlement in the same conditions, the bioactivity of compounds **2** and **12** against mussel plantigrades can be associated with the presence of the sulfate groups. However, other sulfated compounds did not show notable activity. These results show that the nature of the scaffold plays a role in placing sulfate groups in favorable position for the activity: (i) while xanthone **6** was highly active, a structure related xanthone (compound **7**) in which the *O*-linkage was replaced by a triazole linkage, was not active; (ii) while flavonoid **2** was highly active, other three flavonoids (compounds **1**, **3**, and **4**) did not show notable activity, showing that sulfate groups at positions 5, 7, 3′, and 4′ on the flavonoid scaffold were crucial for the anti-settlement activity on this macrofouling species; and (iii) since compound **13**, with an alkylcarboxy group was much less potent than compound **12**, which contains an arylcarboxy group, the degree of acidity of the carboxylic group seems to influence the activity.

From the three promising compounds, a good level of effectiveness (defined by an EC_50_ value < 25 μg.mL^−1^) was only achieved for compound **12**. Considering the effectiveness, compound **12** (EC_50_ = 8.4 μg.mL^−1^) was recognized to have the highest potential as effective AF agent. When comparing the structures of three most promising compounds, it is reasonable to hypothesize that a benzoic acid scaffold (compound **12**) is associated with a more interesting AF activity. In fact, the importance of a benzoic acid moiety in the AF activity was previously observed^32^,^33^. It is interesting to point out that the hit compound in this study, gallic acid persulfate (**12**) is structurally-related to a previously described naturally-occurring AF agent, the zosteric acid, a metabolite from the sea grass *Zostera marina*. Zosteric acid has been found to prevent the attachment of bacteria^14^,^15^,^16^,^34^,^35^, fungus^17^,^36^ and higher-order organisms^37^ at nontoxic concentrations. The AF capability of zosteric acid was firstly attributed to the sulfate ester group^14^. More recently, the anti-biofilm activity of zosteric acid against *E. coli* was proven to be related to the cinnamic acid scaffold^12^,^32^.

When compared with the commercial eco-friendly AF agent ECONEA^®^, sulfated compounds presented lower levels of effectiveness, however have evidenced more potential as ecofriendly AF agents considering the nontoxic effects found.

The specific AF molecular targets and the mechanisms of action of booster biocides currently in used in marine paints remain largely unknown. AF compounds described in literature with known molecular targets mainly act on ion channels, enzymes, adhesive production and neurotransmission blocking^6^. Therefore, in this study, *in vitro* quantification of two enzymes (acetylcholinesterase and tyrosinase) having a metabolic role in the settlement of macrofouling organisms was performed to assess a possible mechanism of action for the promising synthesized compounds (S2). Acetylcholinesterase (AChE) has been suggested as being involved in the settlement of marine organisms, with AChE inhibitors being found to inhibit larval settlement^38^,^39^, while DOPA-containing adhesive plaques of mussels are thought to be produced by the action of tyrosinase on a protein precursor^40^. Nevertheless, no effect was observed in the activity of these enzymes indicating that at least these two pathways seem to remain unchanged after exposure to the synthesized compounds and the observed bioactivity seems to be not linked to these functions inhibition.

Considering the antibacterial activity, sulfated compounds did not present particular effectiveness, as results indicate that only compounds **2** and **4** had the ability to inhibit bacterial growth (less than 30% at all the concentrations tested) of one of the bacterial strains each (*V. harveyi* and *H. aquamarina*, respectively). The fact that low antibacterial activity was found and no consistent growth inhibition results were observed among all the tested bacterial strains seems to give an indication of sulfated compounds anti-settlement mode of action. In fact, the mode of action playing in *M. galloprovincialis* plantigrades seems to be organism-specific, more related with plantigrades metabolic pathways, rather than with the biofouling ecological succession cascade, which starts with the colonization of biofilms^41^. On the other hand, these results put in evidence the low toxicity of these sulfated compounds towards different bacterial strains, enhancing their potential as AF agents.

Concerning general ecotoxicity assessment, the standard lethality *A. salina* bioassay revealed that none of these promising compounds exerted significant lethal effects at the higher concentrations tested in anti-settlement assays (25, 50 and 250 μM), showing the low toxicity potential of these synthesized compounds. The nontoxic nature of the promising compounds **2**, **6**, and **12** was also confirmed by the lack of light radiation inhibition on the *V. fischeri* luminescent assay. Adding to the low toxicity potential, the bioactive sulfated compounds **2**, **6**, and **12**, are highly water soluble, suggesting also a low bioaccumulation potential. Existing measurements of persistence, bioaccumulation and biodegradation are a cumbersome and expensive process, and thus not applied in the early stages of the product discovery and development. Nevertheless, *in silico* screening tools for the search for “environmentally-benign” antifouling biocides provide a valuable resource in early decision-making when applied with discretion^42^. Thus, log *K*_ow_ (octanol-water partition coefficient) were calculated for the most promising compounds (compounds **2**, **6**, and **12**) and compared with those calculated for some marketed AF agents and in contrast to the latter, compounds **2**, **6**, and **12** were shown to have low potential for bioaccumulation. In conclusion, new synthetic AF scaffolds were disclosed in this work, namely a polysulfated flavonoid, xanthone, and phenolic acid, with effective anti-settlement response towards *M. galloprovincialis* plantigrades, high seawater solubility, and general low toxicity and low potential for bioaccumulation. The viable synthetic process can predict their easy scale-up and future commercialization. Thus these results give important contributions for the future development of new environmental friendly AF coatings.

## Material and Methods

All experiments were conducted in accordance with ethical guidelines of the European Union Council (Directive 2010/63/EU) and the Portuguese Agricultural Ministry (Portaria nr. 1005/92 of 23 October 2010) for the protection of animals used for experimental and other scientific purposes.

The starting materials for sulfation, diosmin (D 3525), rutin (R 2303), tri-hydroxyethylrutin (91950), mangiferin (M 3547), *trans*-resveratrol 3-*β*-D-glucopyranoside (572691), salicin (S 0625), 4-methyl 7-hydroxycoumarin 7-*β*-D-glucopyranoside (M 3633), 4-methyl 7-hydroxycoumarin 7-*β*-D-galactopyranoside (M 1633), gallic acid (D 3525), and chlorogenic acid (25700) were purchased from Sigma-Aldrich, Spain. The starting materials of compounds **4**, **6**, and **7**, 3,7-di(*β*-D-glucopyranosyl)flavone, 3,6-bis(*β*-D-glucopyranosyl)xanthone, and 3,6-bis(1-(1-(*β*-D-galactopyranosyl)-1*H*-1,2,3-triazole-4-yl)methoxy)xanthone, respectively, were obtained following described procedures^43^,^44^ and their syntheses will be described elsewhere.

Melting points were obtained in a Köfler microscope and are uncorrected. Infrared (IR) spectra were recorded on a Perkin Elmer 257 in KBr. Nuclear magnetic resonance (NMR) spectra were taken in DMSO-d_6_ at room temperature, on Bruker DRX 300 instrument. Chemical shifts are expressed in δ (ppm) values relative to tetramethylsilane (TMS). High-resolution mass spectrometry (HRMS) results were obtained in CACTI services, Vigo, Spain.

High performance liquid chromatography with Diode-Array Detection (HPLC-DAD) analyses were carried out on a System SMI Pump Series II (Gloucester, UK) equipped with a Rheodyne 7125 injector fitted with a 20 μL loop, a TSP-UV6000LP detector, and a Chromquest for Windows NT integrator and using a C-18 Nucleosil column (5 μm, 250 × 4.6 mm I.D.), from Macherey-Nagel (Düren, Germany); acetonitrile was of HPLC grade from Merck. HPLC ultrapure water was generated by a Milli-Q system (Millipore, Bedford, MA, USA). The mobile phase used was water with tetrabutylammonium bromide (TBAB, 25 mM) and acetonitrile (38:62) at a constant flow rate of 1.0 mL.min^−1^. The mobile phase was filtered (45 μm) and degassed for 15 min in an ultrasonic bath before use.

### Syntheses and structure elucidation of sulfated compounds

**Figure 1:**
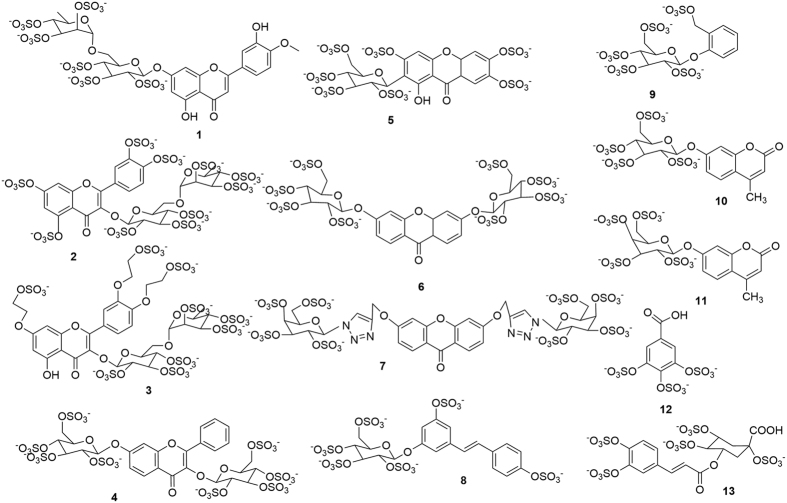
Sulfated compounds chemical structures. **1**, diosmin 2″,2″′,3″,3″′,4″,4″′-hexasulfate; **2**, rutin 2″,2″′,3′,3″,3″′,4′,4″,4″′,7-nonasulfate; **3**, 3″,4″-bis(2-sulfate ethoxy)-7-(2-sulfate ethoxy)-rutin 2″,2″′,3′,3″′,4′,4″′,7-sulfate; **4**, 3,7-di(*β*-D-glucopyranosyl)flavone persulfate; **5**, mangiferin 2′,3,3′,4′,6,6′,7-heptasulfate; **6**, 3,6-bis(*β*-D-glucopyranosyl)xanthone persulfate; **7**, 3,6-bis(1-(1-(*β*-D-galatopyranosyl)-1*H*-1,2,3-triazole-4-yl)methoxy)xanthone persulfate; **8**, *trans*-resveratrol 3-*β*-D-glucopyranoside persulfate; **9**, salicin persulfate; **10**, 4-methylumbelliferyl 7-*β*-D-glucopyranoside persulfate; **11**, 4-methylumbelliferyl 7-*β*-D-galactopyranoside persulfate; **12**, gallic acid persulfate; **13**, chlorogenic acid persulfate.

**Figure 2:**
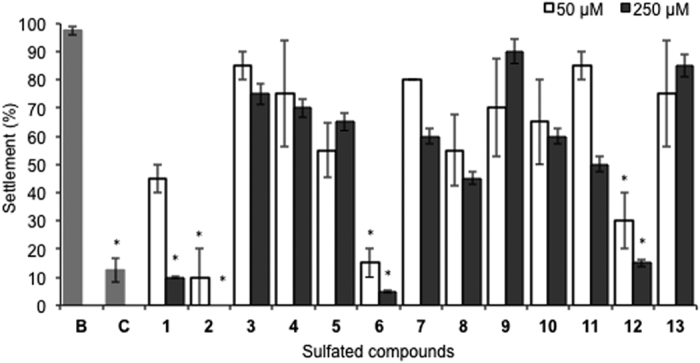
Anti-settlement activity of sulfated compounds 1–13 towards plantigrades of the mussel *Mytilus galloprovincialis*. *Indicates significant differences (*p* < 0.05, Dunnett test) against the negative control (B: ultra-pure water); CuSO_4_ at 5 μM was used as positive control (C).

**Figure 3:**
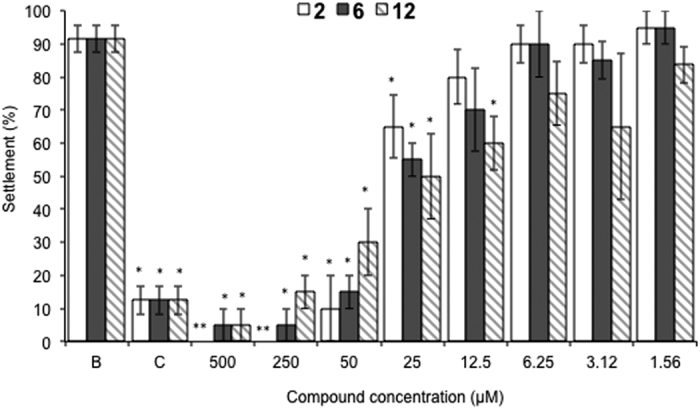
Concentration-response of anti-settlement activity of the promising AF compounds 2, 6, and 12 towards plantigrades of the mussel *Mytilus galloprovincialis*. *Indicates significant differences at *p* < 0.05 (Dunnett test) ** at *p* < 0.001 (Dunnett test), against the negative control (B: ultra-pure water); CuSO_4_ at 5 μM was used as positive control (C).

**Figure 4:**
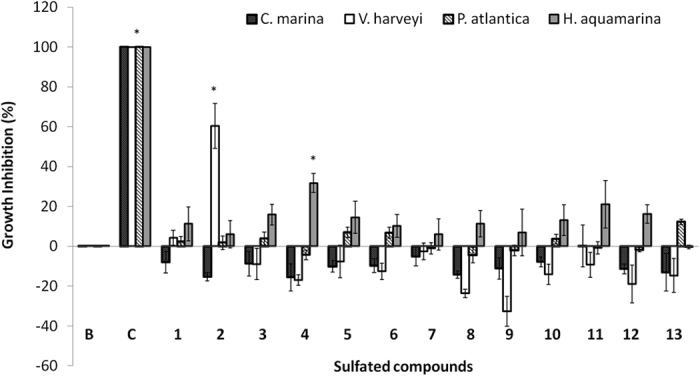
Antibacterial activity of sulfated compounds 1–13 towards four biofilm-forming marine bacteria *Cobetia marina, Vibrio harveyi, Pseudoalteromonas atlantica* and *Halomonas aquamarina*. *Indicates significant diferences (*p* < 0.05, Dunnett test) against the negative control (B: ultra-pure water); penicilin-streptomycin-neomycin stabilized solution was used as positive control (C).

**Figure 5:**
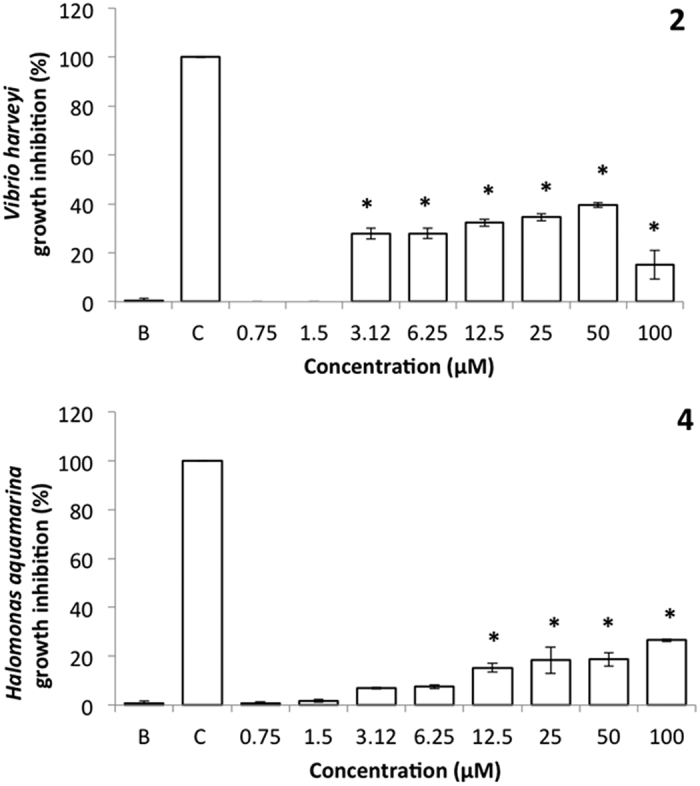
Concentration-response of antibacterial activity of the promising AF compounds 2 and 4 towards *Vibrio harveyi* and *Halomonas aquamarina*, respectively. *Indicates significant differences at *p* < 0.05 (Dunnett test) against the negative control (B: ultra-pure water); penicilin-streptomycin-neomycin stabilized solution was used as positive control (C).

The following sulfated derivatives were synthesized according to previously described procedures (Fig. 1): diosmin hexasulfate (**1**), rutin persulfate (**2**), 3″,4″-bis(2-sulfate ethoxy)-7-(2-sulfate ethoxy)-rutin 2″,2″′,3′, 3″′,4′,4″′,7-sulfate (**3**), mangiferin 2′,3,3′,4′,6,6′,7-heptasulfate (**5**), 3,6-bis(*β*-D-glucopyranosyl)xanthone persulfate (**6**), *trans*-resveratrol 3-*β*-D-glucopyranoside persulfate (**8**), salicin persulfate (**9**), 4-methyl 7-hydroxycoumarin 7-*β*-D-glucopyranoside persulfate (**10**), gallic acid persulfate (**12**), and chlorogenic acid persulfate (**13**)^45^,^46^. Synthesis of new sulfated derivatives, compounds **4**, **7**, and **11**, was achieved by sulfation of 3,7-di(*β*-D-glucopyranosyl)flavone, 3,6-bis(1-(1-(*β*-D-galactopyranosyl)-1*H*-1,2,3-triazole-4-yl)methoxy)xanthone, and 4-methyl 7-hydroxycoumarin 7-*β*-D-galactopyranoside, respectively, in microwave (MW) with triethylamine-sulfur trioxide adduct (8–10 equiv/OH) in dimethylacetamide. Generally, the reaction vessel was sealed, and the mixture was kept stirring and heated for 1 h at 100 °C under MW irradiation (2 cycles). After cooling, the mixture was poured into acetone (200 mL) under basic conditions (triethylamine until pH 8) and left at 4 °C for 24 h. The crude oil formed was washed with acetone and ether and then dissolved in aqueous solution of 30% sodium acetate (5 mL). The suspension was added dropwise in ethanol to precipitate the sodium salt of the sulfated derivative. In both syntheses of compounds **4** and **7**, each solid obtained was further purified from other salts (monitored by IR) using a Spectra/Por 6 regenerated cellulose MWCO 1000. The purity of the investigated compounds was determined by HPLC-DAD analysis.

#### 3,7-Di(2,3,4,6-tetrasulfate-β-glucopyranosyl)flavone (4)

Orange solid, mp 215–218 °C (methanol). IR (KBr, cm^−1^) υmax: 1627, 1066, 803. ^1^H NMR (DMSO-*d*_*6*_, 300.13 MHz) δ: 8.27 (2 H, d, *J* = 8.7 Hz, H-2′, H-6′), 8.07 (1 H, d, *J* = 8.7 Hz, H-5), 7.58–7.48 (3 H, m, H-3′, H-4′, H-5′), 7.30 (1 H, d, *J* = 2.2 Hz, H-8), 7.13 (1 H, dd, *J* = 8.7 and 2.2 Hz, H-6), 6.02 (1 H, d, *J* = 4.4 Hz, H-1″), 5.66 (1 H, d, *J* = 2.9 Hz, H-1″′), 4.76 (1 H, d, *J* = 4.4 Hz, H-2″), 4.72 (1 H, d, *J* = 4.0 Hz, H2″′), 4.65 (1 H, d, *J* = 1.7 Hz, H-3″), 4.62 (1 H, d, *J* = 2.4 Hz, H-3″′), 4.47 (1 H, t, *J* = 4.1 Hz, H-4″), 4.41 (1 H, t, *J* = 3.1 Hz, H-4″′), 4.20 (1 H, m, H-5″), 4.04–3.90 (5 H, m, H-5″′ and H-6″a, H-6″b, H-6″′a, H-6″′b). ^13^C NMR (DMSO- *d*_*6*_, 75.47 MHz) δ: 173.0 (C-4), 161.5 (C-9), 156.0 (C-7), 154.8 (C-1′), 143.6 (C-2), 136.5 (C-3), 130.4 (C-4′), 129.2 (C-2′, C-6′), 128.6 (C-3′, 5′), 126.8 (C-5), 118.1 (C-10), 115.5 (C-6), 103.3 (C-8), 98.8 (C-1″), 98.7 (C-1″′), 80.6 (C-2″), 76.2 (C-2″′), 76.1 (C-5″), 75.5 (C-5″′), 74.8 (C-3″), 74.2 (C-3″′), 72.0 (C-4″), 71.1 (C-4″′), 67.3–66.9 (C-6″, C-6″′). HRMS (ESI^+^) m/z calcd for C_27_H_22_Na_9_O_38_S_8_ [M + Na] 1416.66285, found 1416.66256.

#### 3,6-Bis(1-(1-(2,3,4,6-tetrasulfate-β-D-galactopyranosyl)-1H-1,2,3-triazole-4-yl)methoxy)xanthone (7)

White solid, mp 205–208 °C (methanol). IR (KBr, cm^−1^) υmax: 1260, 1052, 805. ^1^H NMR (DMSO-*d*_*6*_, 300.13 MHz) δ: 8.24 (1 H, s, triazole), 8.08 (1 H, d, *J* = 8.9 Hz, H-1/H-8), 7.37 (1 H, brd, H-4/H-5), 7.18 (1 H, brdd, H-2/H-7), 5.92 (1 H, d, *J* = 8.5 Hz, H-1′), 5.35 (2 H, s, OC*H*_*2*_), 4.90 (1 H, s, H-2′), 4.83 (1 H, t, *J* = 8.8 Hz, H-4′), 4.53–4.49 (1 H, dd, *J* = 1.9 and 9.5, H-3′), 4.18 (1 H, *J* = 8.2, H -5′), 4.03–3.77 (2 H, m, H-6′a, H-6′b). ^13^C NMR (DMSO-*d*_*6*_, 75.47 MHz) δ: 174.2 (C-9), 163.5 (C-3, C-6), 157.5 (C-4a, C-10a), 141.2 (CH = *C* triazole), 127.4 (C-1, C-8), 124.4 (*C*H = C triazole), 115.1 (C-8a, C-9a), 113.9 (C-2, C-7), 101.5 (C-4, C-5), 86.3 (C-1′), 75.5 (C-5′), 75.0 (C-3′), 73.6 (C-2′), 72.1 (C-4′), 66.3 (C-6′), 62.0 (O*CH*_*2*_). HRMS (ESI^+^) m/z calcd for C_31_H_26_N_6_Na_10_O_38_S_8_ [M + H] 787.85091, found 787.85080.

#### 4-Methyl-7-[(2S,3R,4R,5S,6R)-3,4,5-trisulfate-6-(methylsulfate)oxan-2-yl] oxychromen-2-one (11)

Pallid-yellow solid, mp > 340 °C (ethanol). IR (KBr, cm^−1^) υmax: 1617, 1257, 1032, 847. ^1^H NMR (DMSO-*d*_*6*_, 300.13 MHz) δ: 7.73 (1 H, d, *J* = 8.7 Hz, H-8), 7.04 (1 H, dd, *J* = 8.8 and 1.5 Hz, H-6), 6.93 (1 H, d, *J* = 1.5 Hz, H-5), 6.24 (1 H, s, H-3), 5.57 (1 H, d, *J* = 2.9 Hz, H-1′), 4.59 (2 H, m, H-2′ and H-4′), 4.42 (1 H, m, H3′), 4.00–3.88 (2 H, m, H-6′a/H-6′b), 1.16 (3 H, s, 4-C*H*_*3*_). ^13^C NMR (DMSO-*d*_*6*_, 75.47 MHz) δ: 160.2 (C-2), 160.2 (C-7), 154.4 (C-9), 153.6 (C-9/C-4), 113.6 (C-6), 103.6 (C-5), 111.8 (C-3), 114.3 (C10), 126.7 (C-8), 98.4 (C-1′), 75.2 (C-5′), 74.1 (C-2′/C4′), 73.9 (C-2′/C4′), 70.9 (C-3′), 67.1 (C-6′), 18.3 (4-*C*H_3_). HRMS (ESI^+^) m/z calcd for C_16_H_14_Na_5_O_20_S_4_ [M + Na] 768.84443, found 768.84443.

### *Mytilus galloprovincialis* plantigrades collection and processing

*M. galloprovincialis* juvenile (0.5 cm shell length approximately) aggregates were collected from mussel beds from the intertidal rocky shore, during low neap tides at Memória beach, Matosinhos, Portugal (41°13′59″N; 8°43′28″W), and immediately transported to the laboratory. *M. galloprovincialis* plantigrade post-larvae (0.5–2 mm) were screened among the small mussel aggregates in a binocular magnifier (Olympus SZX2-ILLT), gently washed with filtered seawater to remove organic debris and sand particles and isolated in a petri dish with filtered seawater immediately before the bioassays.

### Antifouling bioassays

For the screening bioassay, competent *M. galloprovincialis* plantigrades (showing exploring behavior, ie, moving their foot searching for the appropriate substrate to settle) were selected and exposed to the series of sulfated compounds **1**–**13** at two concentrations (250 and 50 μM) in 24-well microplates for 15 h, at 18 ± 1 °C, in the darkness. Bioassays time frame was selected based on preliminary bioassays to ensure that the response measured is reflecting the compounds effect. Test solutions were prepared in filtered seawater (previously treated by UV light, and carbon filters and mechanically filtered with 0.45 μM filter before use) and obtained by dilution of the compounds stock solutions (50 mM) in ultra-pure water. Four well replicates were used per condition with five plantigrades per well and 2.5 mL of test solution. A negative control, with ultra-pure water was included in all bioassays, as well as a positive control with 5 μM CuSO_4_ (a potent AF agent). At the end of the exposure period, the anti-settlement activity was determined by the presence/absence of byssal threads produced by each individual efficiently attached for all the conditions tested.

Compounds that have showed anti-settlement effectiveness at the concentration of 50 μM were selected for further testing at higher and lower successive concentrations (500, 250, 100, 50, 25, 12.5, 6.25, 3.12 and 1.56 μM) for the determination of the semi-maximum response concentrations that inhibited 50% larval settlement (EC_50_) and the median lethal dose (LC_50_). The commercial eco-friendly AF agent ECONEA^®^ was also tested in the same experimental conditions for bioassay validation and to obtain comparable data regarding effectiveness and toxicity of sulfated compounds.

The seawater stability of the three most promising compounds **2**, **6**, and **12** in the time period (15 h) and temperature (18 °C) of the AF assay was evaluated by HPLC-DAD. Compounds were analyzed at 250 μM (final concentration) in filtered seawater (45 μm). Incubations were performed in glass HPLC vials and three independent samples plus respective blank (filtered seawater) and controls (time zero - freshly prepared solutions of compounds) were analyzed.

To test the hypothesis that the exerted antifouling activity is due to the molecular modification by sulfation, chemical precursors of promising AF compounds **2** and **12**, rutin (**14**), and gallic acid (**15**), respectively, were also tested for AF activity at two concentrations (250 and 50 μM), at the same bioassay conditions as previously described, except for the negative control that in this case includes 0.1% DMSO, considering the low water solubility of the chemical precursors.

### *In vitro* determination of acetylcholinesterase and tyrosinase activities

Acetylcholinesterase and tyrosinase inhibition were tested as potential AF mechanisms of action of compounds **2**, **6**, and **12.**

The potential of compounds **2**, **6** and **12** to inhibit acetylcholinesterases activity was evaluated using *Electrophorus electric* acetylcholinesterase Type V-S (SIGMA C2888, E.C. 3.1.1.7), according to Ellman *et al*.^47^ with some modifications^48^,^49^. Briefly, reaction solution containing phosphate buffer 1 M pH 7.2, dithiobisnitrobenzoate (DTNB) 10 mM (acid dithiobisnitrobenzoate and sodium hydrogen carbonate in phosphate buffer) and acetylcholine iodide 0.075 M was added to pure acetylcholinesterase enzyme (0.25 U/mL) and each test sulfated compound (final concentration of 50 μM) in quadruplicate. The optical density was measured at 412 nm in a microplate reader during 5 min at 25 °C. A negative control (B) with ultra-pure water and a positive control (C+) with 20 mM eserine also included.

Tyrosinase inhibition assays were conducted using *Agaricus bisporus* tyrosinase (EC 1.14.18.1) according to Adhikari *et al*.^50^ with slight modifications. The enzymatic reaction follows the catalytic conversion of L-Dopa to dopaquinone and the formation of dopachrome by measuring the absorvance at 475 nm. Briefly, tyrosinase (25 U/mL) is added to 50 mM fosfate buffer pH 6.5 and the sulfated compound at 50 μM. The enzymatic activity was triggered by the addition of L-dopa (25 mM). Kojic acid was included as positive control and ultra-pure water as negative control.

### Antibacterial activity

Antibacterial activities were evaluated against four strains of marine biofilm-forming bacteria *Cobetia marina* CECT 4278, *Vibrio harveyi* CECT 525, *Halomonas aquamarina* CECT 5000, and *Pseudoalteromonas atlantica* CECT 570, all from Spanish Type Culture Collection (CECT). The bacteria were inoculated and incubated for 24 h at 26 °C in marine broth (Difco) at an initial density of 0.1 (OD_600_) in 96 well flat-bottom microtiter plates. Sulfated compounds stock solutions were prepared in ultra-pure water. For the initial screening, a final concentration of 12.5 μM was used in the bioassays, given the higher sensitivity of the bacteria when compared to mussel plantigrades. Bacterial growth inhibition in the presence of the test compounds was measured in quadriplicate by reading of optical density at 600 nm using a microplate reader (Biotek Synergy HT, Vermont, USA). Marine broth with ultra-pure water and penicilin-streptomycin-neomycin stabilized solution were used as negative (B) and positive (C) controls, respectively.

Compounds that showed significant inhibitory activity at this initial screening were selected for further bioassays. For the determination of maximal inhibitory concentrations (IC) a serial dilution of the stock solution was used to obtain final concentrations from 100 μM to 0.75 μM, the same range applied in antifouling bioassays plus one lower concentration, and the same procedure was applied.

### *Artemia salina* ecotoxicity assay

Selected sulfated compounds with AF potential **2**, **6**, and **12** were tested for general toxicity to nontarget species using the brine shrimp (*Artemia salina*) nauplii lethality test^51^. Briefly, *A. salina* eggs were allowed to hatch in nutrient–enriched seawater as nauplii for approximately 48 h at 25 °C. Toxicity tests were performed in the darkness in 96-wells microplates with eight replicates per condition and 15–20 nauplii per well. Serial diluted test solutions of the most promising sulfated AF compounds (25, 50, and 250 μM) were prepared in filtered seawater. K_2_Cr_2_O_7_ (13.6 μM) was included as positive control and ultra-pure water as negative control. Percentage of mortality was determined at 48 h of exposure.

### Luminescent *Vibrio fischeri* ecotoxicity assay (ISO11348)

Luminescent *Vibrio fischeri* ecotoxicity assessment (ISO11348) was performed by IK4 TEKNIKER accordingly to the EU hazard assessment of substances and European Ecolabel (ISO 113482). Luminescent *Vibrio fischeri* bacteria test (NRRL-B-11177) was used as a standard test to evaluate the ecotoxicity of the most promising AF compounds. *Vibrio fischeri* bacteria from HACH-LANGE GmbH were grown in laboratory in standard conditions according to guidelines and exposed to a dilution serial of each compound (1000, 500, 250, 125, 62.5 mg/L). After 5, 15 and 30 min of exposure, the light emitted as a by-product of *Vibrio fischeri* cellular respiration was measured at 490 nm. Any inhibition of cellular activity results in a decreased rate of respiration and a corresponding decrease in the rate of luminescence. The decrease of bacterial luminescence measured after 5, 15 or 30 min of exposition was used as test endpoint. Luminescence was measured using a LUMIStox photometer from DR LANGE, after a contact time of 15 min and 30 min at 15 ± 1 °C, taking into account a correction factor, *fk*, which is a measure of intensity change of control samples during the exposure time.

The pH of all samples was within the interval 6.0–8.5. A 2% solution of sodium chloride (NaCl) in deionized water (20 g/L) was used as dilution medium and K_2_Cr_2_O_7_ 11.3_ _μg.mL^−1^) was used as positive control.

### *In silico* evaluation of bioaccumulation potential

KOWWIN™ v1.68 (log octanol-water partition coefficient calculation program)^52^ developed by Syracuse Research Cooperation jointly with the Environmental Protection Agency (EPA) was used for *in silico* calculation of log *K*ow (octanol-water partition coefficient) in order to evaluate the bioaccumulation potential of compounds **2**, **6**, and **12** when compared to other AF active principles in use. Compounds are considered potentially bioaccumulative if the log Kow (octanol-water partition coefficient) is higher than 3.

### Data analysis

Data from the anti-settlement and antibacterial screenings were analyzed using a one-way analysis of variance (ANOVA) followed by a Dunnett test against the control (*p* < 0.05). Semi-maximum response concentration that inhibited 50% larval settlement (EC_50_), semi-maximum response concentration that inhibited 20% bacterial growth (EC_20_) and the median lethal dose (LC_50_) for each bioactive compound were assessed using Probit regression analysis. Pearson Goodness-of-fit (Chi-Square) significance was considered at *p* < 0.05 for all analyses, and 95% lower and upper confidence limits [95% LCL; UCL] were presented. Therapeutic ratio (LC_50/_EC_50_) was used to evaluate the effectiveness vs toxicity of compounds^31^,^53^,^54^. The software IBM SPSS Statistics 21 was used for statistical analysis.

## Additional Information

**How to cite this article:** Almeida, J. R. *et al*. Antifouling potential of Nature-inspired sulfated compounds. *Sci. Rep.* **7**, 42424; doi: 10.1038/srep42424 (2017).

**Publisher's note:** Springer Nature remains neutral with regard to jurisdictional claims in published maps and institutional affiliations.

## Supplementary Material

Supplementary Information

## Figures and Tables

**Table 1:** Antifouling effectiveness *versus* toxicity of sulfated compounds 2, 6, and 12 and the commercial AF compound ECONEA^®^ towards the anti-settlement of mussel plantigrades.

Compound	EC_50_ (μM; μg.mL^−1^)	LC_50_ (μM)	LC_50_/EC_50_
**2**	22.59 (95% CI: 15.50–35.36); 36.84	>500	>22.13
**6**	23.19 (95% CI: 15.70–35.40); 31.74	>500	>21.56
**12**	17.65 (95% CI: 9.46–32.86); 8.4	>500	>26.61
ECONEA^®^	4.012 (95% CI: 0.38–9.54); 1.40	107.78 (95% CI: 83.61–144.04)	26.86

EC_50_, minimum concentration that inhibited 50% of larval settlement; LC_50,_ the median lethal dose; LC_50_/EC_50_, therapeutic ratio. Note: reference values for EC_50_ < 25 μg.mL^−1^ (U.S. Navy program) and therapeutic ratio (LC_50_/EC_50_) higher than 15.

**Table 2:** Bacterial growth inhibition activities of compounds 2 and 4.

Compound	EC_20_ (μM; μg.mL^−1^)	
	*V. harveyi*	*H. aquamarina*
**2**	7.69 (95% CI: 2.3–17.4); 12.54	>100
**4**	>100	42.3 (95% CI: 31.2–60.9); 51.22

EC_20_, minimum concentration that inhibited 20% of bacterial growth.
