# Supplementary material for: Antifouling potential of Nature-inspired sulfated compounds
Source: Sci Rep. 2017 Feb 13;7:42424. doi: 10.1038/srep42424 (PMC5304334; doi:10.1038/srep42424)
Supplement: Supplementary Information [file srep42424-s1.pdf]

Antifouling potential of Nature-inspired sulfated compounds

Joana R. Almeida[1], Marta Correia-da-Silva[1,2]*, Emília Sousa[1,2], Jorge Antunes[1,3], Madalena Pinto[1,2], Vitor Vasconcelos[1,3], Isabel Cunha[1]

[1]CIIMAR/CIMAR - Interdisciplinary Centre of Marine and Environmental Research, University of Porto. Terminal de Cruzeiros do Porto de Leixões
Avenida General Norton de Matos P 4450-208 Matosinhos, Portugal.

[2]Laboratory of Organic and Pharmaceutical Chemistry, Department of Chemical Sciences, Faculty of Pharmacy. University of Porto, Rua Jorge Viterbo Ferreira, 228, 4050-313 Porto, Portugal.

[3]Department of Biology, Faculty of Sciences, University of Porto. Rua do Campo Alegre, P 4069-007 Porto, Portugal.

*Corresponding author: m_correiadasilva@ff.up.pt

SUPPLEMENTARY INFORMATION

S1.

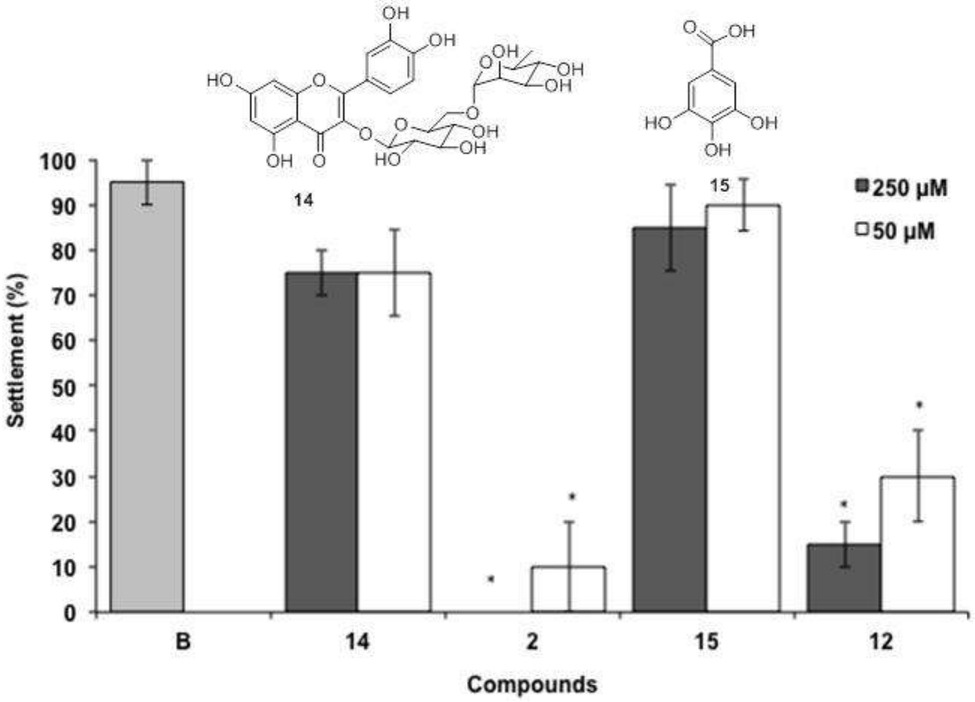

Anti-settlement activity of the two chemical percursors **14** and **15** (250 and 50 µM) and sulfated derivatives (**2** and **12**), towards plantigrade larvae of the mussel *Mytilus galloprovincialis*. B: filtered seawater with 0.01% DMSO.

S2.

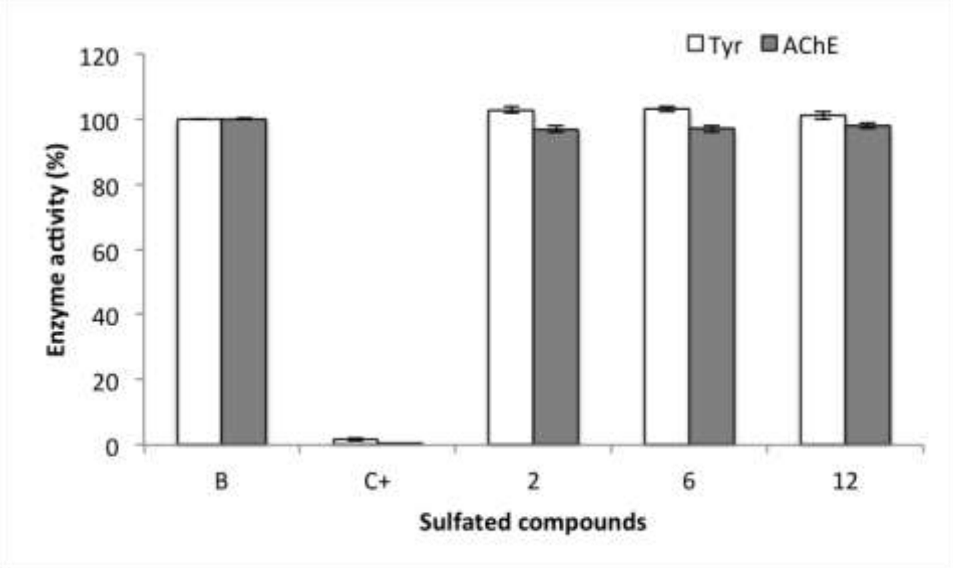

In vitro acetylcholinesterase (AChE) and tyrosinase (Tyr) activities in the presence of the three promising sulfated compounds (**2, 6** and **12**). B: ultra-pure water control; C+: positive control with eserine (20 mM) and kojic acid (20 mg/mL) for AChE and Tyr assays, respectively.

S3.

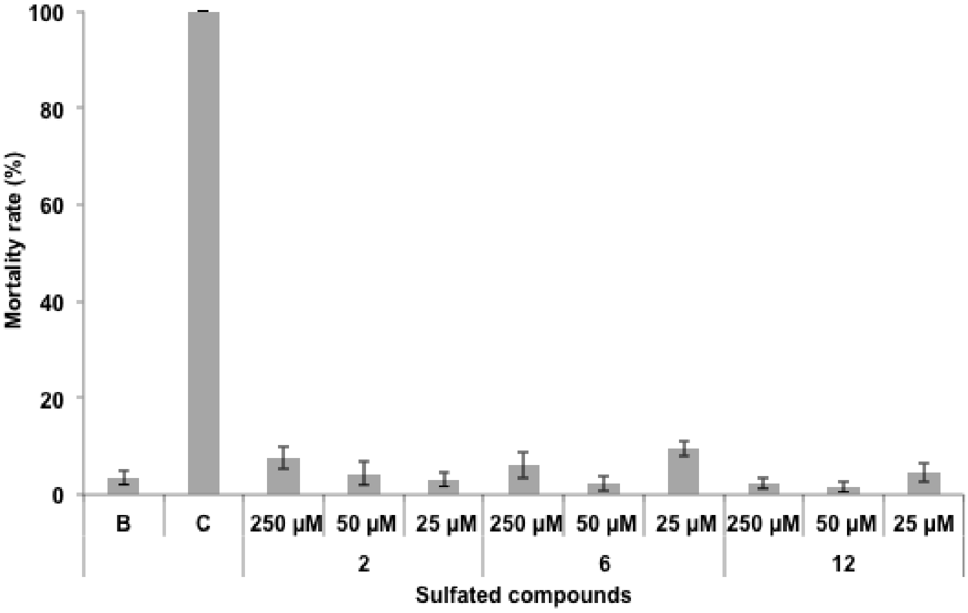

Mortality rate of *Artemia salina* nauplii after 48 h of exposure to the promising AF compounds **2**, **6**, and **12** (250, 50, and 25 µM). B: filtered seawater. $K_2Cr_2O_7$ at 13.6 µM was used as positive control (C).

S4. Growth inhibition activities of compounds **2**, **6**, and **12** towards the luminescent bacteria *Vibrio fischeri*.

Compound	EC_{50} (µg.mL^{-1})	Initial Concentration of tested sample (µg.mL^{-1})
**2**	$EC_{50\ 15min}$: >1000	2000
	$EC_{50\ 30min}$: >1000	
**6**	$EC_{50\ 15min}$: >1000	2000
	$EC_{50\ 30min}$: >1000	
**12**	$EC_{50\ 15min}$: >1000	2000
	$EC_{50\ 30\ min}$: >1000	
$K_2Cr_2O_7$	$EC_{50\ 30min}$: 5.67	-
	$EC_{80\ 30min}$: 16.73	

$K_2Cr_2O_7$ (Potassium dichromate) was used as positive control

S5. Calculated octanol water partition coefficient (Log Kow) for compounds **2**, **6**, and **12** in comparison with other recognised AF agents.

Octanol water partition coefficient	**2**	**6**	**12**	TBTO	Sea-nine	Econea®
Log Kow	-30.26	-26.11	-7.02	3.56*	3.59*	4.69*

TBTO= tributyltin oxide
*Log Kow higher than 3 means potentially bioaccumulative.
